# JAK inhibitors attenuate hyperactivation of nonswitched memory B cells in rheumatoid arthritis patients in remission

**DOI:** 10.1186/s13075-024-03374-x

**Published:** 2024-07-17

**Authors:** Jing Luo, Jing Zhang, Bomiao Ju, Yanhua Wang, Nan Hu, Qian Li, Qianyun Xu, Dan Pu, Zhiming Hao, Yongwei Huo, Xiaohong Lv, Lan He

**Affiliations:** 1 Department of Rheumatology and Immunology, The First Affiliated Hospital of Xi’an Jiaotong University, Xi’an, Shaanxi 710061 China; 2 Department of Human Anatomy and Histology and Embryology, School of Basic Medical Sciences, Xi’an Jiaotong University Health Science Center, Xi’an, Shaanxi 710061 China

**Keywords:** Rheumatoid arthritis, Janus kinase inhibitors, B-cell subpopulations, CD40

## Abstract

**Objective:**

To investigate the distribution and activation of B-cell subpopulations in rheumatoid arthritis (RA) patients treated with Janus kinase inhibitors (JAKis) and to analyze their correlation with disease remission.

**Methods:**

Peripheral blood samples were collected from 23 adult healthy controls and 58 RA patients, 31 of whom were treated with JAKis and assessed during a 24-month follow-up. The number of peripheral B-cell subpopulations (including naive B cells, nonswitched memory B (NSMB) cells, switched memory B cells, and double-negative B cells), their activation, and phosphorylation of SYK and AKT upon B-cell receptor (BCR) stimulation in each population were analyzed by flow cytometry.

**Results:**

Compared with that in healthy controls, the frequency of NSMB cells was significantly lower in new-onset untreated RA patients. However, expression of CD40, CD80, CD95, CD21^low^ and pAKT significantly increased in these NSMB cells. Additionally, the number of NSMB cells correlated negatively with DAS28-ESR and IgG and IgA levels in these patients; expression of CD80, CD95 and CD21^low^ on NSMB cells correlated positively with DAS28-ESR and IgG and IgA levels. After treatment with JAKis, the serum IgG concentration significantly decreased in RA patients in remission, but CD40, CD95 and pAKT levels in NSMB cells significantly decreased.

**Conclusion:**

RA patients present different B-cell subpopulations, in which the frequency of NSMB cells is negatively associated with disease activity. However, treatment with JAKis can inhibit activation of NSMB cells, restore the balance of kinase phosphorylation, and facilitate disease remission in RA patients.

## Introduction

Rheumatoid arthritis (RA) is a chronic inflammatory autoimmune disorder characterized by persistent synovial inflammation, bone and cartilage erosion, and joint destruction. Autoantibodies are valuable markers for RA diagnosis, classification and disease prognosis [1]. B-cell depletion therapy with rituximab has reinforced the important role of B cells in RA [2]. The functions of B cells, including antigen presentation, cytokine secretion and autoantibody production, are all related to the pathogenesis of RA [3]. In many RA patients, synovial extrafollicular germinal centers develop, with B cells playing an intimate role in local inflammation and the generation of memory B cells and plasma cells [4]. Recently, Joo et al. quantified all subsets of B-cell precursors in the bone marrow and splenic B cells in murine RA and demonstrated a severe reduction in pre-B cells and immature B cells in the bone marrow [5]. These findings indicate the critical roles of B cells in the development of RA.

Human B cells can be divided into four main subsets based on their differential expression of immunoglobulin IgD (IgD) and CD27: IgD + CD27- naive B (NAVB), IgD + CD27 + nonswitched memory B (NSMB), IgD-CD27 + switched memory B (SMB) and IgD-CD27- double-negative B (DNB) cells [6]. In our previous study, we found a reduction in NSMB cells in new-onset diagnosed RA patients, which correlated negatively with the serum erythrocyte sedimentation rate (ESR) and immunoglobulin G (lgG) levels [7]. Moura et al. reported that samples from very early-stage RA patients had a significantly decreased frequency of circulating NSMB cells [8]. Additionally, the frequencies of these B cells correlated negatively with tender joint count (TJC), swollen joint count (SJC), and anti-CCP antibody (ACPA) levels [9]. Moreover, SMB cells in the peripheral blood of RA patients express more receptor activator of nuclear factor-κb ligand (RANKL) and activated bone-resorbing osteoclasts, which reveals the importance of B cells in bone homeostasis and their likely contribution to joint destruction in RA [10].

Janus kinase inhibitors (JAKis) are innovative and effective medical drugs for treating RA. JAKis can be taken orally to improve medication compliance. JAKis can inhibit intracellular signaling controlled by numerous cytokines implicated in the disease process of RA. Recent evidence has suggested that tofacitinib impairs in vitro plasmablast development and immunoglobulin secretion from naive B cells, increases the proportions and absolute numbers of B cells during the first 8–12 weeks, and reduces the number of B cells after 24 weeks of treatment in thirteen RA patients [11]. However, few studies have focused on alterations in B-cell subpopulations and activation in RA patients during JAKi treatment. The primary objective of this study was to investigate the distribution, activation and phosphorylation status of SYK and AKT upon B-cell receptor (BCR) stimulation in each population of B cells from RA patients and to analyze the relationship of these factors with disease activity after JAKi treatment.

## Materials and methods

### Subjects

This was a prospective, single-center cohort study that included 81 subjects, 58 of whom were RA patients in the Department of Rheumatology and Immunology, First Affiliated Hospital of Xi’an Jiaotong University, Shaanxi, China; the other 23 were age- and sex-matched healthy volunteers. Of the RA patients, 31 received JAKi treatment from August 2019 to June 2022 according to the following inclusion criteria: (i) aged more than 18 years and (ii) fulfilled the American College of Rheumatology 2010 criteria for RA [12]. There was no any type of blinding in treatment allocation.The exclusion criteria were definitely diagnosed infection, risk of major adverse cardiovascular events (MACE) and cancer or end-stage disease. The risk factor for MACE are cardiovascular disease that is significantly unstable or not effectively treated, such as myocardial infarction, malignant arrhythmia, unstable angina pectoris, or uncontrolled high blood pressure with medication within the last 6 months. “End-stage” disease referred to end-stage renal disease (ESRD), liver cirrhosis.

The untreated group included patients who are newly diagnosed, and who received oral or local topical non-steroidal anti-inflammatory drugs discontinuously. The patients had never received glucocorticoids, conventional disease-modifying anti-rheumatic drugs(csDMARDs), targeted synthetic disease-modifying anti-rheumatic drugs or biological disease-modifying anti-rheumatic drugs.The patients treated with JAKis were subclassified based on visit time at month 0 (T0, *n* = 31), 3 (T3, *n* = 14), 6 (T6, *n* = 13), 12 (T12, *n* = 18) or 24 (T24, *n* = 12). In our 24-month study, patients were not consistent across each time point, and the consecutive and complete clinical data of 10 patients were available during entire follow-up visit time.Their demographic and clinical features were obtained at baseline. Clinical symptoms, laboratory data, medications and 28-joint Disease Activity Score with erythrocyte sedimentation rate (DAS28-ESR) were assessed at each follow-up point.The laboratory data collected included rheumatoid factor (RF) and ACPA levels, hypersensitive C-reactive protein (hs-CRP) levels, ESR, and serum IgG, serum immunoglobulin M (IgM), and serum immunoglobulin A (IgA) levels. This study was conducted in accordance with the Declaration of Helsinki, the protocol was approved by the Ethics Committee of the First Affiliated Hospital of Xi’an Jiaotong University, and all participants provided written informed consent.

### Sample collection and flow cytometry

Flow cytometry was used for lymphocyte immunophenotyping. Peripheral blood mononuclear cells (PBMCs) were isolated by density-gradient centrifugation using Ficoll-Paque TM PLUS (Cytiva, Sweden AB) according to standard protocols.

The following antibodies were used for BCR stimulation: CD19-APC (BD Biosciences, 555,415), IgD-FITC (BD Biosciences, 555,778), CD27-PE (BD Biosciences, 555,441), CD95-PerCP-C5.5 (BioLegend, 305,629), CD40-APC-Cy7 (BioLegend, 334,323), CD21-BV421 (BD Biosciences, 566,260), CD80-BV510 (BD Biosciences, 563,084), pSYK-PerCP-Cy5.5 (BioLegend, 683,710), pAKT-BV421 (BD Biosciences, 562,599), and anti-human IgG, IgM (H + L chain) (BioLegend, 397,302).

B-cell activation markers (including CD40, CD80, CD95 and CD21^low^) were assessed in the 81 subjects. After staining with antibodies, cells were assessed with CytoScan (Beckman Coulter, USA), and the data were analyzed with CytoSex.

To measure BCR-induced signaling in this study, PBMCs were first incubated in RPMI medium at 37 °C for 30 min. Afterward, staining with APC-labeled human monoclonal antibodies against CD19, IgD and CD27 for another 30 min at 37 °C was performed. Next, the PBMCs were stimulated with F(ab’)2 anti-IgM (at a concentration of 2 µg/mL) for 5 min. Following stimulation, the cells were fixed using BD CytofixTM Fixation Buffer at a volume equal to the initial staining volume for 10 min at 4 °C. After fixation, the PBMCs were centrifuged at 400 × g for 3 min and washed twice with BD PhoSlowTM Perm/Wash buffer. The cells were then stained with antibodies specific for phosphorylated spleen tyrosine kinase (pSYK) and phosphorylated serine/threonine kinase (pAKT) for 1.5 h at 4 °C in the dark. Subsequently, the PBMCs were washed again with Perm/Wash buffer and resuspended in a volume of 200 µl of Perm/Wash buffer for analysis by flow cytometry.

### Statistical analysis

Statistical analysis was performed with SPSS software 22.0 (SPSS, Inc., Chicago, IL, USA). Descriptive analysis (calculations of averages, proportions, or rates) was also conducted. The Shapiro‒Wilk test and Levene test were used to evaluate the normality and homogeneity of variance, respectively. The significance of differences in means between groups was assessed by Student’s t test or the Mann‒Whitney U test; differences in correlations were assessed by Spearman analysis.We used Spearman correlation analysis to analyze correlations between disease activity parameters and alteration of NSMB cells frequency, activation markers and BCR signals in our study. The reason for choosing this analysis is that the variables involved did not satisfied conditions for using Pearson correlation analysis (continuous data, normal distribution, and linear relationship). P values less than 0.05 were considered to indicate statistical significance.

## Results

### Clinical and demographic characteristics of the RA patients

The clinical and demographic characteristics of the patients with RA and healthy controls are shown in Table 1. DAS28-ESR < 2.6 was considered to indicate remission. Of the 58 RA patients, 27 had new-onset disease, and 31 had remission status after JAKi treatment (29 patients received tofacitinib at a dose of 5 mg twice daily and 2 received baricitinib at a dose of 4 mg once daily). There was no difference in sex or median age between the two groups (*P* > 0.05). ESR was greater in untreated RA patients than in patients in remission after JAKi treatment (40 mm/h vs. 12 mm/h, *P* < 0.001). Consistently, the same results were observed for hs-CRP (4.83 mg/L vs. 0.76 mg/L, *P* < 0.001), IL-6 (14.1 pg/mL vs. 4.99 pg/mL, *P* = 0.008) and IgG (15.5 g/L vs. 13.1 g/L, *P* < 0.001). However, no difference in IgA or IgM levels was observed between the two groups.

**Table 1:** Demographic and characteristics of RA patients in different cohorts

Parameters	Healthy(*n* = 23)	Untreated group(*n* = 27)	JAKi remission group(*n* = 31)	*P* value
Age, years	41(39.60)	45(40,61)	43(35,57)	0.092
Gender, female(n,%)	21(91.3%)	24(88.9%)	28(90.3%)	0.799
Duration of disease, years		1(0.2–2.5)	4(2–10)	0.008
Swollen joint count(0–28)		5 (2–7)	0(0–1)	0.000
Tender joint count(0–28)		6 (3–10)	1(0–1)	0.000
Pain, VAS 0–10 cm		4.5(2.8–7.3)	0.8(0-1.1)	0.001
HAQ-DI		1.625(0.625–1.875)	0(0-0.125)	0.000
DAS28-ESR		5.27(3.96-6.00)	2.10(1.90–2.85)	0.000
ESR, mm/1 h		40(30–66)	12(6–20)	0.001
hs-CRP, mg/L		4.83(1.91–7.49)	0.76(0.28–1.96)	0.001
IL-6,pg/mL		14.1(5.1–27.7)	4.99(1.5–14.1)	0.008
RF-positive(n,%)		21(77.7%)	24(77.4%)	0.777
ACPA-positive(n,%)		22(81.5%)	25(80.6%)	0.798
IgG, g/L		15.5(13.3–18.7)	13.1(11.4–14.6)	0.000
IgA, g/L		2.86(2.01–3.37)	2.51(1.96–3.42)	0.483
IgM, g/L		1.29(0.88–1.75)	1.11(0.68–1.71)	0.641
Medication(n,%)				
Tofacitinib + MTX			10(32.3%)	
Tofacitinb + LEF			8(25.8%)	
Tofacitinib + IGU			5(16.1%)	
Tofacitinib + TNFi			4(12.9%)	
Baricitinib + LEF			2(6.45%)	
Tofacitinib + HCQ			2(6.45%)	

All data are presented as the median (QR), MTX, methotrexate; LEF, leflunomide; IGU, Iguratimod; TNFi, adalimumab, etanercept; HCQ, hydroxychloroquine

### Effects of JAKis on B-cell subpopulations, surface activation markers and BCR signals

**Fig. 1:**
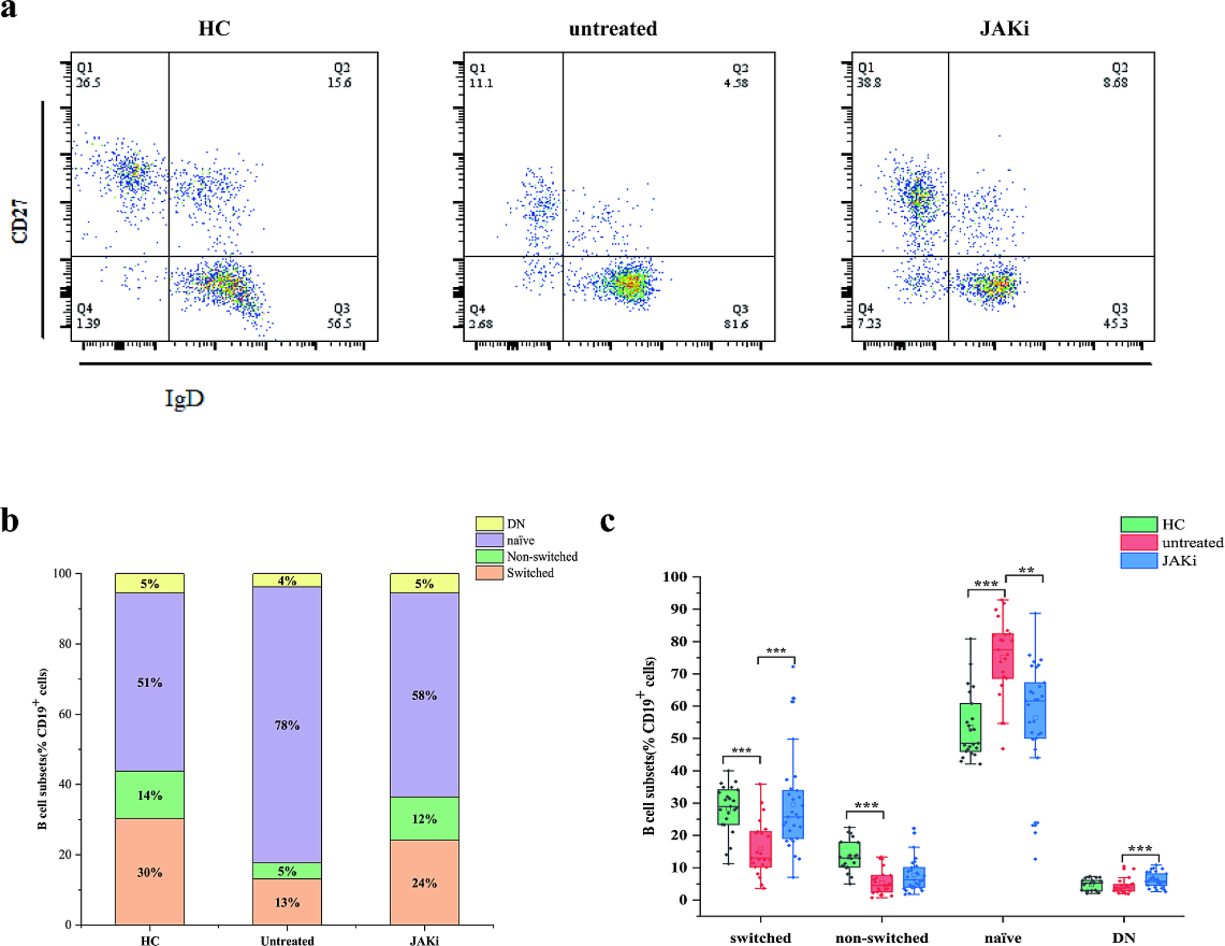
Distribution of B-cell subpopulations in new-onset RA patients (*n* = 27) and RA patients in remission after JAKi treatment (*n* = 31) compared to healthy controls (*n* = 23). **(a)** The gating strategy for identifying B-cell subpopulations based on the expression of IgD and CD27, which included switched memory (CD27 + IgD-), nonswitched memory (CD27 + IgD+), naive (CD27-IgD+), and DN (CD27-IgD-) B cells. **(b)** Diverse composition of B-cell subpopulations in the peripheral blood of healthy controls and patients with untreated RA and RA remission after JAKi treatment. **(c)** Comparative analysis of switched, nonswitched, naive and DNB cells was conducted to evaluate the distribution of the B-cell subpopulation in the peripheral blood of healthy controls and untreated patients and patients with remission of RA after JAKi treatment. Data are shown in box-whisker plots with individual dots, where boxes represent the 25th to 75th percentiles and the lines within the boxes represent the medians. P values were determined with Mann-Whitney U test using GraphPad Prism 6 (**P < 0.01, ***P < 0.001)

As shown in Fig. 1a-c, frequencies of CD27 + IgD + NSMB cells and SMB cells were significantly lower in new-onset RA patients than in healthy controls (both *P* < 0.001), but the frequency of NAVB cells was greater (*P* < 0.001). There was no significant increase in DNB cells in new-onset RA patients. After JAKi treatment, the number of NAVB cells decreased (*P* < 0.01), but proportions of SMB cells (*P* < 0.001) and NSMB cells (*P* = 0.065) gradually increased. The proportion of DNB cells also increased (*P* < 0.001).

**Fig. 2:**
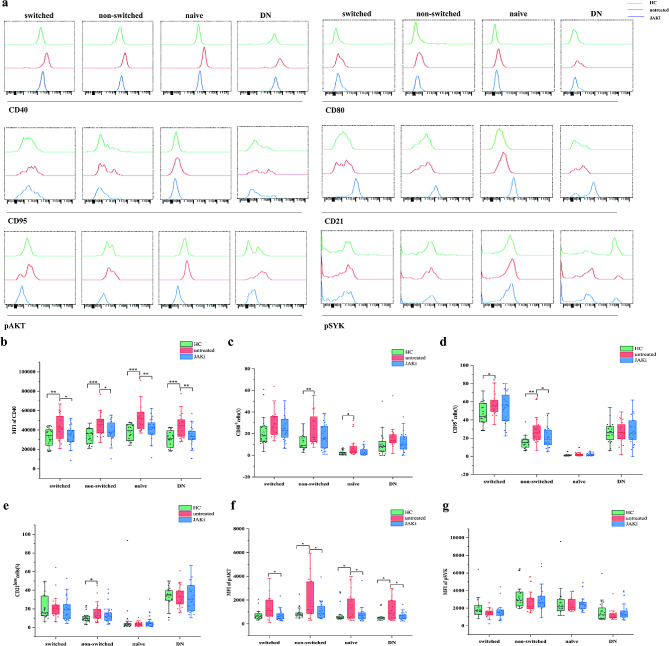
Surface activation marker and BCR signalling analysis of the B-cell subpopulation in new-onset RA patients (*n* = 27) and RA patients in remission after JAKi treatment (*n* = 31) compared to healthy controls (*n* = 23). **(a)** A representative histogram demonstrating the MFI of CD40; proportions of CD80, CD95, and CD21^low^; and the MFI of AKT and SYK phosphorylation 5 min after stimulation with an antibody against BCR in healthy controls and untreated patients and patients with remission of RA after JAKi treatment. Comparative analysis of **(b)** the MFI of CD40, **(c)** proportions of CD80, **(d)** proportions of CD95, **(e)** proportions of CD21^low^, **(f)** the MFI of pAKT, and **(g)** the MFI of pSYK on switched, nonswitched, naive and DNB cells was conducted in the peripheral blood of healthy controls and patients with untreated RA and RA remission after JAKi treatment. Data are shown in box-whisker plots with individual dots, where boxes represent the 25th to 75th percentiles and the lines within the boxes represent the medians. P values were determined with Mann-Whitney U test using GraphPad Prism 6 (*P < 0.05, **P < 0.01, ***P < 0.001)

Moreover, expression of CD40, CD80, CD95, CD21^low^, and pAKT on NSMB cells was significantly increased in new-onset RA patients (*P* < 0.05). For RA patients in remission, expression of CD40, CD95 and pAKT on NSMB cells was significantly decreased after JAKi treatment (*P* < 0.05). Additionally, CD40 and pAKT levels increased in NAVB, SMB and DNB cells from new-onset RA patients (*P* < 0.01). CD80 expression on NAVB cells significantly increased (*P* = 0.011), and CD95 expression increased in SMB cells (*P* = 0.015). After JAKi treatment, expression of CD40 and pAKT in NAVB, SMB and DNB cells decreased (*P* < 0.05) (Fig. 2a-g).

### Dynamic changes in B-cell subpopulations, activation markers and BCR signals during 24 months of JAKi treatment

**Fig. 3:**
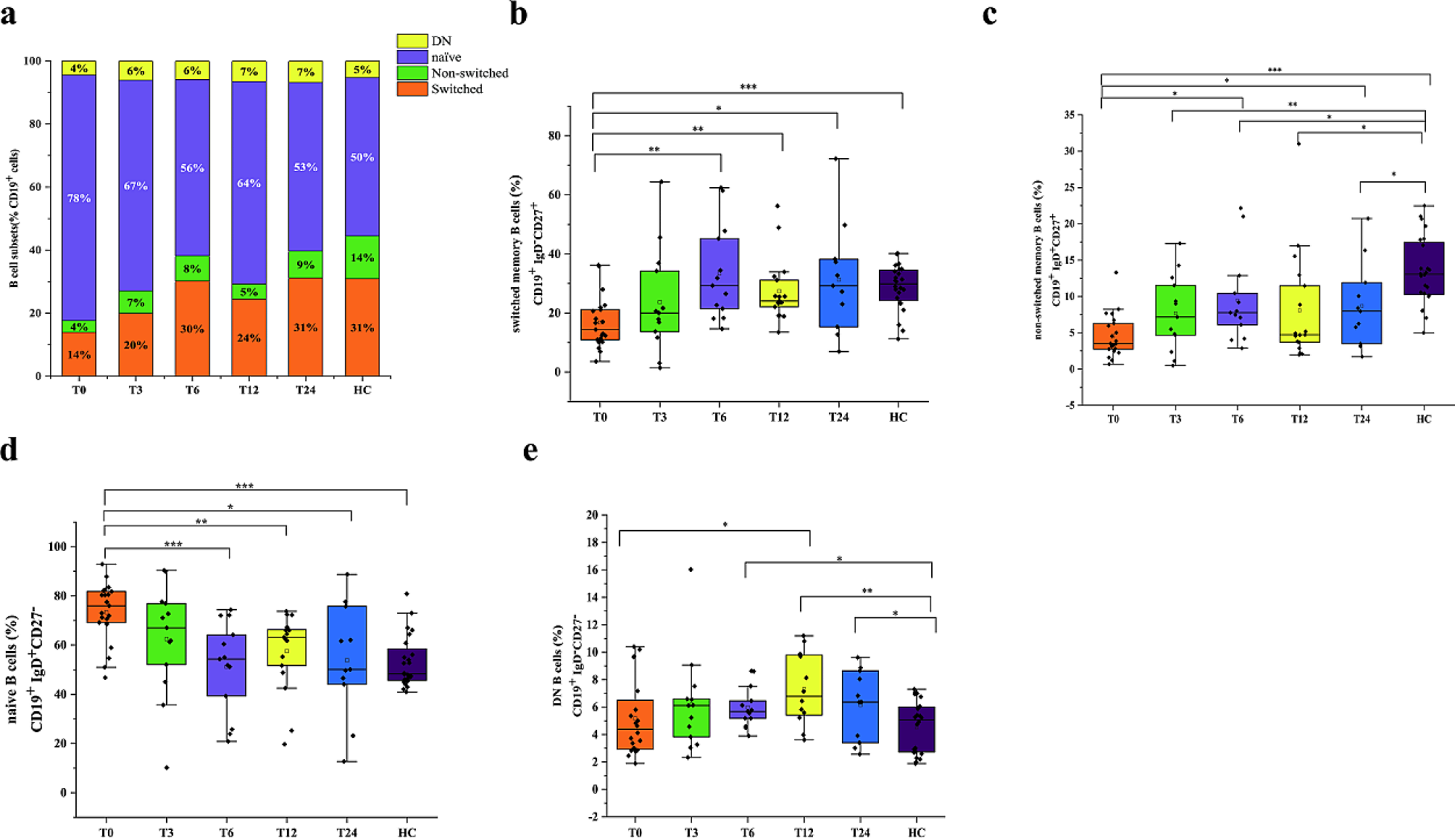
Dynamic changes in B-cell subpopulations during 24 months of JAKi treatment. **(a)** Diverse composition of B-cell subpopulations in the peripheral blood of RA patients at each visit (T0, T3, T6, T12 and T24) during JAKi treatment and in healthy controls. **(b)** Dynamic changes in switched memory B cells. **(c)** Dynamic changes in nonswitched memory B cells. **(d)** Dynamic changes in naive B cells. **(e)** Dynamic changes in DNB cells during 24 months of JAKi treatment

The patients treated with JAKis were subclassified based on visit time at month 0 (T0, *n* = 31), 3 (T3, *n* = 14), 6 (T6, *n* = 13), 12 (T12, *n* = 18) or 24 (T24, *n* = 12). The demographic and clinical features of these patients are shown in Table 2. Compared to those at baseline, ESR and DAS28-ESR started to decrease gradually after T3. Patients maintained low disease activity and remission from T6. IgA and IgG were significantly lower at T6 and at T12, respectively (*P* < 0.05). RF and ACPA did not significantly differ between the groups. As shown in Fig. 3a, the distribution of B-cell subpopulations changed during 24 months of JAKi treatment. The main variation was an increased proportion of switched and nonswitched memory B cells (Fig. 3b **and** Fig. 3c) and a decreased proportion of naive B cells (Fig. 3d). This frequency change fluctuated at T12 and returned to equilibrium at T24. Additionally, the DNB cell proportion increased at T12 (Fig. 3e).

**Table 2:** Clinical characteristics of patients treated with JAKis during the 24-month follow-up

Parameters	T0(*n* = 31)	T3(*n* = 14)	T6(*n* = 13)	T12(*n* = 18)	T24(*n* = 12)
Age, years	49(35,63)	42(38,57)	43(38,56)	50(43,64)	44(34,55)
Gender, female(n,%)	24(85.7%)	13(92.9%)	10(76.9%)	14(94.3%)	11(91.7%)
Disease duration, months	2(1–8)	3(0.5-8)	1.5(0.7-6)	8(1.2–10.5)	5(3-10.5)
DAS28-ESR	4.6(3.9-6.0)	3.0(2.4–3.5)*	2.9(2.0-3.2)*	2.6(1.9–2.9)*	2.2(1.8–2.8)*
ESR, mm/1 h	44(22–77)	14(7–30)*	12(8–29)*	12(6–18)*	13(6–15)*
RF, IU/mL	84.2(17.3–234)	63(11–140)	98(9.1–1170)	106.5(24.3–268)	74(11.6-440.5)
ACPA, U/mL	359.8(11.9-818.1)	98.5(5.1-447.1)	46.5(0.6–131)	208.9(67.6-433.6)	66.3(4.85–797.8)
IgG, g/L	15.4(11.7–17.5)	15.1(13.6–16.7)	13.5(11.9–15.0)	12.5(10.7–13.7)*	13.4(11.4–15.2)
IgA, g/L	3.1(2.7–4.1)	3.1(2.3–3.9)	2.0(1.7–2.8)*	3.0(2.2–4.1)	2.7(1.9–3.6)
IgM, g/L	1.5(0.9–1.9)	1.3(0.4-2.0)	1.1(0.9–2.2)	1.1(0.7–1.5)	0.9(0.6–1.9)
Medication(n,%)					
GC	1(3.5%)	0	0	0	0
MTX	5(17.8%)	4(28.6%)	4(30.7%)	6(33.3%)	3(25%)
MTX dose, mg/w	12.5(10–15)	9.4(7.5–10)	9.2(7.5–10)	8.1(5–10)	7.5(5–10)
LEF	4(14.3%)	3(21.4%)	2(15.4%)	5(27.7%)	5(41.7%)
IGU	4(14.3%)	2(14.3%)	2(15.4%)	2(11.1%)	3(25%)
TNFi	0	0	0	1(5.5%)	1(8.3%)
Baricitinib	0	0	0	2(11.1%)	2(16.7%)
Tofacitinib	0	14(100%)	13(100%)	16(88.9%)	10(83.3%)

All data are presented as the median (QR). Abbreviations: *P < 0.05

**Fig. 4:**
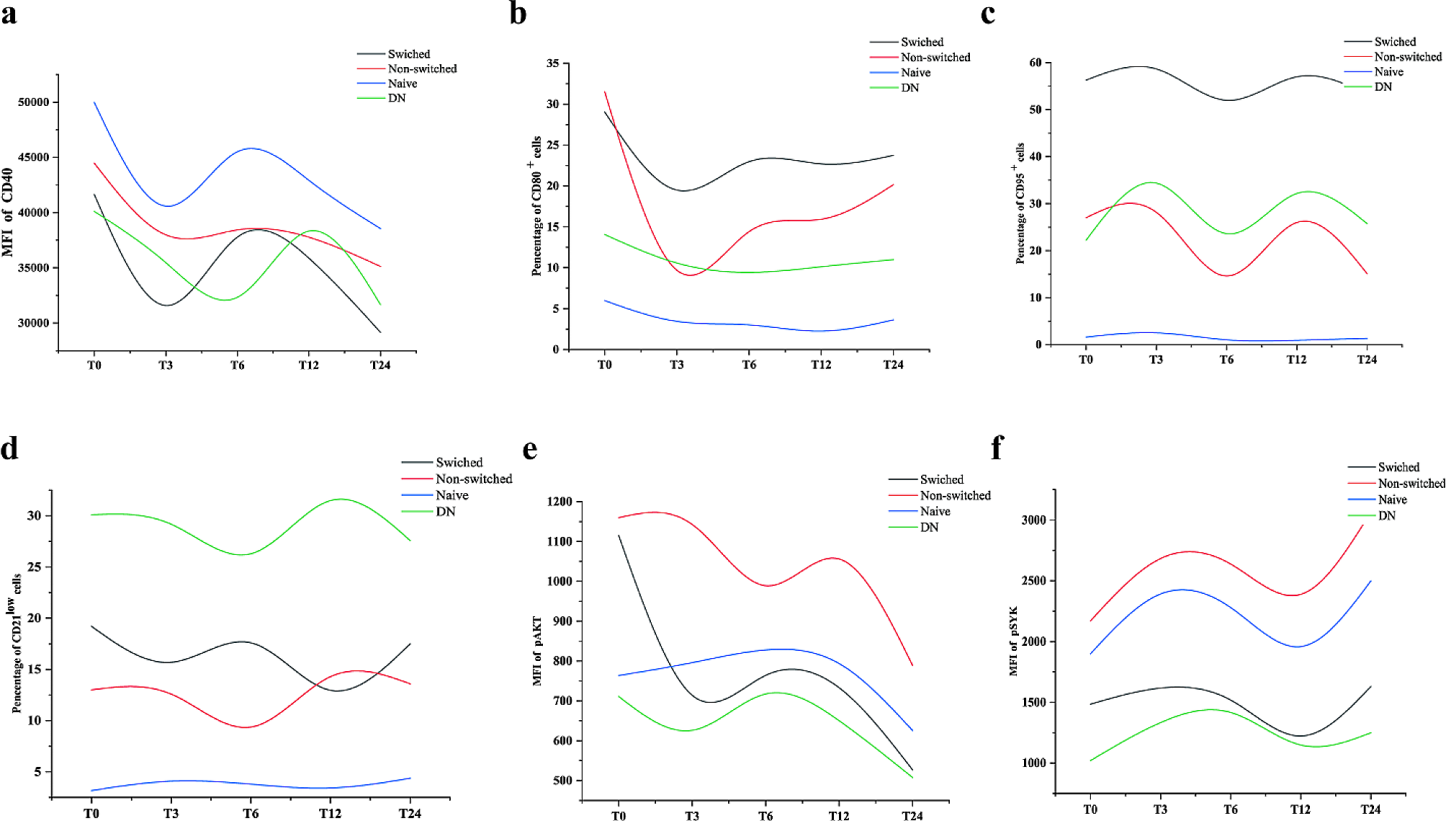
Dynamic changes in activation markers and BCR signals during 24 months of JAKi treatment. **(a)** MFI of CD40; **(b)** proportions of CD80; **(c)** proportions of CD95; **(d)** proportions of CD21^low^ cells on switched, nonswitched, naive and DNB cells; **(e)** MFI of pAKT 5 min after BCR stimulation; **(f)** MFI of pSYK 5 min after BCR stimulation on switched, nonswitched, naive and DNB cells during 24 months of JAKi treatment

B-cell surface activation molecules and BCR signals were differentially expressed in each subgroup. CD40 expression was highest in naive B cells (Fig. 4a), CD95 expression in SMB cells (Fig. 4c), and CD21^low^ expression in DNB cells (Fig. 4d). CD80 and pAKT expression was highest in NSMB cells (Fig. 4b **and** Fig. 4e). During the 2-year period of JAKi treatment, CD40 and CD80 levels on NSMB cells decreased rapidly during the first 3 months, and expression of CD95 on NSMB cells, SMB cells and DN B cells decreased significantly at the 6th month.

### Correlations between clinical parameters and activation markers and BCR signals in NSMB cells

The frequency of NSMB cells correlated negatively with DAS28-ESR and IgG and IgA levels. The frequency of naive B cells correlated positively with DAS28-ESR and the IgG level. MFI of CD40 on NSMB cells correlated positively with hCRP (*r* = 0.324, *p* = 0.007), and CD80 expression on NSMB cells correlated positively with DAS28-ESR (*r* = 0.255, *P* = 0.037). CD95 expression on NSMB cells correlated positively with ESR (*r* = 0.275, *P* = 0.035) and the IgG level (*r* = 0.256, *P* = 0.030). CD21^low^ on NSMBs correlated positively with IgA levels (*r* = 0.256, *P* = 0.030). However, no significant correlation between the ACPA concentration and the B-cell subpopulation distribution was observed(Table 3**)**.

**Table 3:** Correlation analysis of B cells subsets, activation markers on NSMB cells and clinical manifestations

clinical manifestations	SMB(%)		NSMB (%)		DNB (%)		NAVB(%)		CD80+ on NSMB(%)		CD95+ on NSMB(%)		CD21^low^ on NSMB(%)	
	*r*	*p*	*r*	*p*	*r*	*p*	*r*	*p*	*r*	*p*	*r*	*p*	*r*	*p*
DAS28-ESR	-0.190	0.072	-0.254*	0.018	-0.149	0.161	0.231*	0.027	0.255*	0.037	0.163	0.189	0.086	0.490
ESR	-0.247*	0.025	-0.182	0.084	0.148	0.183	0.283*	0.009	0.258*	0.048	0.275*	0.035	0.192	0.145
h-CRP	-0.218	0.053	-0.042	0.714	-0.199	0.077	0.216	0.054	0.177	0.150	0.094	0.447	0.029	0.816
CCP	0.079	0.490	-0.120	0.294	-0.018	0.875	0.026	0.822	-0.045	0.716	-0.091	0.460	-0.003	0.978
RF	0.010	0.929	-0.077	0.508	-0.109	0.350	0.050	0.669	0.113	0.358	0.008	0.950	-0.082	0.509
IgG	-0.109	0.059	-0.209*	0.049	-0.115	0.285	0.232*	0.029	-0.060	0.615	0.256*	0.030	0.009	0.937
IgM	0.166	0.121	0.187	0.079	0.036	0.740	-0.189	0.076	-0.038	0.753	0.101	0.398	-0.051	0.673
IgA	-0.196	0.068	-0.356*	0.012	0.034	0.751	0.158	0.130	-0.016	0.895	0.069	0.566	0.256*	0.030

Bold fond indicates having statistical significance

*P < 0.05

## Discussion

B cells play an important role in the pathogenesis of RA and accumulate in the synovium and form ectopic germinal center, where they may differentiate into antibody-secreting plasma cells [13]. This study was conducted to assess the effects of JAKis on B-cell subpopulations in RA patients. The results showed that JAKis can affect the distribution of B-cell subpopulations and regulate expression of B-cell activation molecules and phosphorylation of key kinases in the BCR signalling pathway and that these changes are associated with disease remission in RA patients.

We found that compared with healthy controls, new-onset untreated RA patients presented an abnormal distribution of B-cell subpopulations, in which the frequency of NSMB cells decreased but the frequency of NAVB cells increased, consistent with the findings of a previous study [14]. After disease remission with JAKi treatment, the frequency of NAVB cells returned to normal level, and the frequency of NSMB cells increased, although it did not reach a normal level. Our study demonstrated that JAKis improved the imbalance in the distribution of peripheral B-cell subpopulations in patients with RA.

Furthermore, we determined that MFI of CD40, the expression of CD80, CD95, and CD21^low^ and the MFI of pAKT after BCR stimulation were increased in NSMB cells from new-onset untreated RA patients. After JAKi treatment, the MFI of CD40, the expression of CD80, CD95, and CD21^low^ and the MFI of pAKT after BCR stimulation of NSMB cells significantly decreased. In our study, the MFI of CD40 on NSMB cells correlated positively with hCRP. In addition, the expression of CD80 on NSMB cells correlated positively with the DAS28-ESR. CD40, a member of the TNF receptor (TNFR) family, is constitutively expressed on the surface of mature B cells. JAK is associated with CD40 and is critical for CD40-mediated induction of gene expression in B cells [15]. Both CD40 and CD80 are essential costimulatory molecules for B-cell proliferation, differentiation and maturation. The increase in the MFI of CD40 and the increase in the expression of CD80 on NSMB cells in active RA patients indicate that these cells are hyperactivated, which might have an enhance antigen presenting activity and are associated with the activation of autoreactive T cells. Tofacitinib can suppress human B-cell activation, differentiation and class switching in vitro study. Our findings revealed that JAKis can inhibit the overactivation of NSMB cells through the downregulation of costimulatory molecules, affect T-B-cell interactions and restore immune homeostasis in patients with RA, improving the clinical outcomes of patients with RA. CD95 (FAS) is a marker of apoptosis induced by cell activation and is involved in the maintenance of immune homeostasis. In our study, CD95 expression on NSMB cells correlated positively with the ESR and the IgG concentration. A decrease in NSMB cells might be induced by enhanced apoptosis. However, we did not perform direct measurement of B-cell apoptosis and cannot draw any firm conclusions. Recently, JAKi treatment was shown to reduce the populations of Fas + naive T cells and effector Th cells [16]. Multiple studies have revealed that CD21^low^ B cells exhibit a proinflammatory phenotype and antigen-presenting capacity in autoimmune disorders [17]. In our study, the frequency of CD21^low^ NSMB cells correlated positively with the IgA concentration. The increase in CD21^low^ on NSMB cells further supports the involvement of these hyperactivated cells in the disease process of RA. JAKis can reduce the CD21^low^ NSMB cells to some extent with disease remission.

JAKis inhibit IL-6 production and the interferon (IFN) signalling pathway through blockade of JAK1 [18]. These cytokines can influence B-cell activity by supporting B-cell survival and regulating BCR signalling [19]. In our study, we found that SYK phosphorylation was decreased and AKT phosphorylation was increased in the B-cell subpopulations of untreated RA patients. The hyperresponsive AKT phenotype appears to permit increased survival of autoreactive B cells. JAKis can partially restore the imbalance between SYK and AKT phosphorylation upon BCR stimulation. These findings of kinase activity imbalance are consistent with evidence available in a previous study [20]. Our results indicate that JAKis can affect AKT phosphorylation in peripheral B cells and restore the imbalance of kinase activity, ameliorate immune disorders and alleviate disease activity in patients with RA.

Notably, the MFI of CD40 was highest on naive B cells, which indicates that the activation of B cells is a common phenomenon in RA.Longitudinal analysis of our cohort revealed that these cytological changes persisted during the 2-year follow-up, although the inhibitory effect of JAKis on B-cell immune responses still needs to be further clarified as soon as the drug is removed. Although we did not observe a significant decrease in RF or ACPA titre during the 2 years of JAKi treatment, the antibody level did not increase further. This finding suggested that the autoreactive response of B cells to autoantigens in RA patients in remission after JAKi treatment was restrained to a certain extent.

Our limitations are that this was not a very strict longitudinal cohort, and the sample size was also small at partial visit time point. Moreover, we did not evaluate the effect of JAKis on a broad range of serum cytokines in patients with RA or reveal immune pathways involved in the mechanism by which JAKis attenuate hyperactivation of nonswitched memory B cells.

In conclusion, there are significant changes in the frequencies and aberrant activation of B-cell subpopulations in RA patients. In particular, NSMB cell frequency and activation are closely associated with disease activity. JAKis can inhibit overactivation of NSMB cells, restore the imbalance of SYK and AKT phosphorylation upon BCR stimulation, and promote the equilibrium of B-cell subpopulation distributions in RA patients. Our findings contribute to a better understanding of the pathophysiology of RA and will facilitate further study of RA therapy.

## Data Availability

No datasets were generated or analysed during the current study.

## Abbreviations

ACPA Anti: Citrullinated peptide antibodies
BCR B: Cell receptor
CRP C: Reactive protein
DNB Double: Negative B cells
DAS28: ESR 28-joint Disease Activity Score with erythrocyte sedimentation rate
ESR: Erythrocyte sedimentation rate
hs-CRP: Hypersensitive C-reactive protein
HCQ: Hydroxychloroquine
IgD: Immunoglobulin D
lgG: Immunoglobulin G
IgM: Immunoglobulin M
IgA: Immunoglobulin A
IGU: Iguratimod
IFN: Interferon
IL-6: Interleukin-6
JAKis: Janus kinase inhibitors
LEF: Leflunomide
MACE: Major adverse cardiovascular events
MTX: Methotrexate
NAVB: Naive B cells
NSMB: Non switched memory B cells
PBMCs: Peripheral blood mononuclear cells
pSYK: Phosphorylated spleen tyrosine kinase
pAKT: Phosphorylated serine/threonine kinase
RANKL: Receptor activator of nuclear factor-κb ligand
RF: Rheumatoid factor
RA: Rheumatoid arthritis
SJC: Swollen joint count
SMB: Switched memory B cells
TJC: Tender joint count
TNFi: Tumor necrosis factor inhibitor
